# Bitter but beneficial: Hop-based feed additives for fighting antimicrobial resistance in poultry

**DOI:** 10.1016/j.psj.2026.107376

**Published:** 2026-07-02

**Authors:** Luisa Kober, Sebastian M. Strauch, Veronika Grassl, Monique Fröhlich Tonet, Nora Hirschmann, Dagmar Fischer, Gilmar S. Erzinger, Kathrin Castiglione

**Affiliations:** a Institute of Bioprocess Engineering, Friedrich-Alexander-Universität Erlangen-Nürnberg, Paul-Gordan-Straße 3, 91052, Erlangen, Germany; b Program in Health and Environment, University of Joinville Region - UNIVILLE, Joinville, 89219-710, SC, Brazil; c Division of Pharmaceutical Technology and Biopharmacy, Friedrich-Alexander-Universität Erlangen-Nürnberg, Cauerstraße 4, 91058, Erlangen, Germany; d FAU NeW – Research Center New Bioactive Compounds, Nikolaus-Fiebiger-Straße 10, 91058, Erlangen, Germany

**Keywords:** Microencapsulation, Solid lipid microspheres, Phytogenic feed additive, Poultry gut microbiota, Hop extracts

## Abstract

Antimicrobial resistance (**AMR**) is an increasing challenge in the global production of poultry, where antibiotics have traditionally been used to promote growth. Phytogenic feed additives (**PFA**s), particularly hop-based ones, could be a sustainable alternative due to their antimicrobial properties. The present study investigated two hop extracts from two sources, which are Type 90 hop pellets derived from fresh hops and residue of same hop pellets that had previously undergone CO_2_ extraction, using a cross-model analysis combining *in vitro* (viability assay/broth dilution assay), *ex ovo* (shell-less hen’s egg test) and *in vivo* tests. Both types of extracts exhibited antimicrobial activity *in vitro* and high biocompatibility in the *ex ovo* model. Notably, the biocompatibility was improved for *ex ovo* setup, compared to *in vitro* cytotoxicity, providing important insights regarding their potential application as PFAs. For *in vivo* studies, hop extracts were microencapsulated as solid lipid microspheres to increase their stability and bioavailability. Feeding animals microencapsulated hop extracts or xanthohumol-rich residue from CO_2_ extraction led to a significant reduction specifically of Gram-negative intestinal bacteria, with no change in animal performance. Overall, two promising products were identified that could potentially be used as a sustainable alternative to antibiotics in poultry production.

## Graphical abstract

**Figure:**
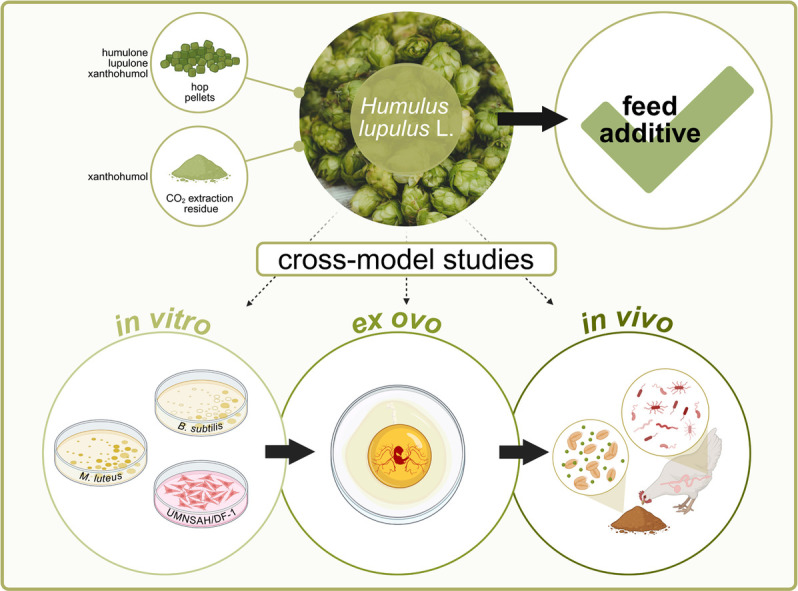

## Introduction

Antimicrobial resistance (**AMR**) is one of the greatest challenges facing global public health and affects both human and veterinary medicine (Jindal et al., 2015). The extensive use of antibiotics in livestock farming is considered a major driver of the selection and spread of resistant bacteria (McEwen and Collignon, 2018). Poultry production in particular plays a central role due to its high production density (Stevenson, 2023) and global significance (Mesquita Souza Saraiva et al., 2022). For many years, antibiotics were used in low doses as antibiotic growth promoters (**AGP**s) to improve feed conversion and animal health (Hardy, 2002). However, growing evidence of a link between this use and the development of AMR has led to far-reaching regulatory measures, including a complete ban on AGPs in all EU countries, the USA, Canada and the UK (Salim et al., 2018). These developments have increased pressure to establish effective, safe, and sustainable alternatives that ensure animal health and performance without contributing to the development of AMR. Poultry production places special demands on management and feeding strategies. The gastrointestinal tract is a key target for interventions, as disturbed intestinal homeostasis is often associated with reduced performance (Kogut and Arsenault, 2016), increased pathogen load, and increased excretion of zoonotic pathogens (Wigley, 2015). In antibiotic-free production systems, feed additives must therefore not only have antimicrobial effects, but also at best contribute to the stabilization of the intestinal microbiome (Sethiya, 2015).

Phytogenic feed additives (**PFA**s), which are derived from herbs, spices and other plant materials, have attracted increased attention in recent years as alternatives to antibiotics. These compounds are distinguished by a broad structural diversity and a wide spectrum of biological activities, including antimicrobial (Aminullah et al., 2025), antioxidant (Abdelli et al., 2021), anti-inflammatory, and immunomodulatory effects (Mahfuz et al., 2021). However, their utilisation in the field of animal nutrition is frequently constrained by factors including instability, rapid degradation in the upper gastrointestinal tract, and sensory disadvantages such as pronounced taste, especially bitterness (Abdelli et al., 2021). Hops, which are an excellent example of phytogenics, are a well-characterised and industrially established source of bioactive compounds. In addition to their traditional use in the brewing industry, hop isolates such as alpha acids (humulone), beta acids (lupulone), and various polyphenolic compounds, especially xanthohumol, have been the focus of extensive research due to their biological effects (Korpelainen and Pietiläinen, 2021). It has been demonstrated that these substances possess antibacterial properties, particularly towards Gram-positive bacteria, in addition to anti-inflammatory and antioxidant characteristics (Gerhäuser, 2005). The mechanisms of action of hop-derived acids include the disruption of bacterial membrane functions and the destruction of the proton gradient (Korpelainen and Pietiläinen, 2021), whereas polyphenols inhibit the replication of microorganisms additionally (Karabín et al., 2016). These properties indicate hop as a promising candidate for use in feeding concepts in poultry production. In previous studies conducted *in vitro*, isolates from fresh hops, including humulone, lupulone, and xanthohumol, exhibited promising results regarding their antibacterial effects, when compared to isomerized hop isolates resulting after the brewing process (Kober and Castiglione, 2025). Analyses of the correlation behaviour of these components also yielded favourable outcomes in combined use compared to their isoforms (Kober et al., 2026). Consequently, it is preferable to utilise material out of fresh hops, for instance hop extracts gained from ethanolic or CO_2_ extraction processes, for the production of PFAs.

Despite the high biological potential of hop-based active ingredients, their direct application in feed poses considerable challenges. These include chemical degradation (Steenackers et al., 2015), interactions with feed components (Abdelli et al., 2021), uneven distribution (McCoy et al., 1994), and negative effects on feed acceptance due to intense bitterness (Abdelli et al., 2021). The success of PFAs therefore depends largely on suitable formulation strategies. In this context, microencapsulation offers a promising approach to protect bioactive substances from external influences, mask sensory disadvantages, and enable targeted release in the gastrointestinal tract. By embedding them in suitable carrier materials, the stability and effectiveness of plant-based ingredients can be significantly improved (Dias et al., 2015; Radrigán et al., 2017). Among the available microencapsulation processes, spray congealing (or spray cooling) is particularly suitable for use in animal nutrition. This solvent-free process is based on the atomization of molten lipid-based carrier materials, which form solid lipid microspheres (**SLM**s) with enclosed active ingredients as they cool. Spray congealing is industrially scalable, compatible with heat-sensitive substances, and allows the production of feed-grade formulations (Rajendran et al., 2022). For hop-based active ingredients, this technology offers the additional advantage of reducing the pronounced bitterness by adding flavouring or sweeteners while enabling delayed release in the intestine, where antimicrobial effects are particularly relevant (Okuro et al., 2013).

The utilisation of microencapsulated hop-based feed additives represents a promising approach to combating antimicrobial resistance in poultry production. Therefore, the aim of this study was to evaluate the efficacy of hop extracts in the context of poultry farming using an integrated experimental approach. Three different SLM batches and residues from CO_2_ extraction were assessed in an *in vivo* setting. Moreover, the effects of hop extracts were further investigated and compared across complementary *in vitro* and *ex ovo* models. By combining these experimental systems, this study addresses the current lack of cross-model evaluations in the literature and provides a comprehensive basis for the development of effective and safe hop-based alternatives to AGPs.

## Materials and methods

### Bacterial strains, media, and culture conditions

*Micrococcus luteus* (DSM 1605) was obtained from DSMZ (German Collection of Microorganisms and Cell Cultures GmbH). It was cultivated on nutrient agar or in nutrient broth (**NB**, DSMZ #1) containing 5 g l^−1^ peptone from casein (Carl Roth, Karlsruhe, GER) and 3 g l^−1^ meat extract (Carl Roth, Karlsruhe, GER) under aerobic conditions at 37°C.

*Bacillus subtilis* (DSM 23778) was obtained from DSMZ (German Collection of Microorganisms and Cell Cultures GmbH). It was cultivated on **TSB** (tryptic soy broth, DSMZ #545) agar plates or in TSB containing 17 g l^−1^ peptone from casein, 3 g l^−1^ peptone from soy, 2.5 g l^−1^ D (+) glucose, 5 g l^−1^ NaCl and 2.5 g l^−1^ K_2_HPO_4_ (Carl Roth, Karlsruhe, GER) under aerobic conditions at 28°C.

For preparation of precultures, 20 mL of the respective medium was inoculated with a colony from a freshly streaked agar plate in 100 mL shaking flasks. Precultures were then incubated for 16 h at either 37°C for *M. luteus* or 30°C for *B. subtilis*, with shaking at 180 rpm and a 50 mm shaking throw (Infors-HT, Bottmingen, CH).

### Chicken cell line, media, and culture conditions

UMNSAH/DF-1 cell line was purchased from Cytion (Eppelheim, GER). The cells were maintained in **DMEM** (Dulbecco’s Modified Eagle Medium, Sigma-Aldrich, St. Louis, USA) containing 10% heat-inactivated **FBS** (fetal bovine serum, Sigma-Aldrich, St. Louis, USA) and cultivated at 37°C and 5% CO_2_ in a humidified atmosphere. Cell lines were serial passaged after being detached with Accutase® solution (Sigma-Aldrich, St. Louis, USA).

### Preparation of ethanolic hop extracts

Ethanolic hop extracts were prepared using an accelerated solvent extractor (**ASE** 350, Dionex, Sunnyvale, USA). Ground hop pellets (brauen.de, Dresden, GER) or residue from CO₂ extraction (Hopsteiner, Mainburg, GER), both of type Herkules (2024 harvest, 19.2% alpha acid content), were used as sample. Extraction was conducted using 80% ethanol/20% H₂O (v/v) mixtures at 100°C. Ethanol is a widely employed solvent for the extraction of plant material and is also relevant for food and feed applications due to its food-grade quality (Cravotto et al., 2023; Magalhães et al., 2007). The dissolution of less hydrophobic components, such as polyphenols, is achieved by the use of hydroethanolic mixtures (Sultana et al., 2009). Moreover, it has been demonstrated that elevated temperatures are also associated with enhanced extraction efficiency due to higher mass transfer rates (Mustafa and Turner, 2011). 500 mg of sample was placed in extraction cells (V = 22 mL) with cellulose filters (Thermo Fisher Scientific, Waltham, USA), according to manual instructions (Thermo Scientific, 2011). The extraction cells were filled with solvent, before static extraction was performed for 30 minutes at a pressure of 100 bar. N₂ was then used to purge the solvent from the cells into 60 mL collecting vials, which were sealed with **PTFE** septa (polytetrafluoroethylene, Thermo Fisher Scientific, Waltham, USA). To avoid contamination and carry-over of extracts, the system was rinsed with solvent between extractions. Additionally, a blank sample (empty extraction cell) was prepared to exclude solvent effects in later analysis.

### Preparation of aqueous hop extracts

After the extraction process, the solvent was removed using a centrifugal evaporator (SPD131DDA, Thermo Fisher Scientific, Waltham, USA) at room temperature and pressure of 10^−3^ bar. The dried extracts were weighed to determine the biomass (**BM**), after which they were stored at −20°C until further preparation. Ultimately, the dried extracts were resolubilized to a final concentration of 20 mg_BM_ mL^−1^ with 2% ethanol (v/v) and 98% of the respective medium for later application (see Table 1). Blank samples were prepared analogously to the sample with the highest biomass. Two different variants of DMEM were used to evaluate the *in vitro* cytotoxic properties. As the aqueous hop extract needs to be as colourless as possible for *ex ovo* application, a medium containing no phenol red (DMEM-Ph, D1145, Sigma-Aldrich) was used. To rule out differences in solubility and therefore cytotoxicity compared to hop extracts prepared in DMEM used for cell culture (DMEM+Ph, D6429, Sigma-Aldrich), it was also tested *in vitro* as well.

**Table 1:** Overview of media used for preparation of aqueous hop extracts. TSB: tryptic soy broth, NB: nutrient broth, DMEM: Dulbecco’s Modified Eagle Medium, Ph: phenol red, FBS: fetal bovine serum, h.i.: heat-inactivated.

Application	Organism	Medium used
*in vitro* antibacterial activity	*Bacillus subtilis*	TSB
*in vitro* antibacterial activity	*Micrococcus luteus*	NB
*in vitro* cytotoxic activity	UMNSAH/DF-1	DMEM+Ph, 10% FBS h.i.
*in vitro* cytotoxic activity	UMNSAH/DF-1	DMEM-Ph, 10% FBS h.i.
*ex ovo* biocompatibility	Egg	DMEM-Ph, 10% FBS h.i.

The resolubilized extracts were placed in an ultrasonic bath for 5 minutes (SONOREX, Bandelin, Berlin, Germany). To remove undissolved biomass and ensure sterility, aqueous extracts were centrifuged (10.000 xg, 10 min), filtered through 0.22 µm **PES** (polyether sulfone) syringe filters (Sarstedt, Nümbrecht, Germany) and stored at 4°C in darkness until further use.

### Broth dilution assay for *in vitro* antibacterial activity

Antibacterial activity of aqueous hop extracts against Gram-positive *M. luteus* and *B. subtilis* was analysed using Broth Dilution Assay (**BDA**). Twofold dilutions of hop extracts were prepared in NB or TSB, ranging from concentrations from 20 mg_BM_ mL^−1^ to 20 µg_BM_ mL^−1^ and transferred to 48-well microtiter plates (Sarstedt, Nuembrecht, GER). Gentamicin sulfate (Carl Roth, Karlsruhe, GER) was used as a positive control. The optical density (**OD**) of a preculture prepared on the previous day was measured at 600 nm in a spectrophotometer (Implen, Muenchen, GER). The inoculum was then diluted with NB or TSB to the desired OD_600nm_ of 0.1 and transferred to all wells (150 µL) except the sterile control, which consists only of medium (300 µL). The ODs of the cultures were measured every hour at 750 nm using a plate reader (Tecan, Maennedorf, CH) for 24 h. In the meantime, the plates were incubated at 37°C (*M. luteus)* or 28°C (*B. subtilis*) without shaking. The chosen wavelength of 750 nm resulted from previous experiments with hop extracts in order to avoid an overlap of the absorption spectrum with chlorophyll (Zepka et al., 2017). The growth rates were obtained from the exponential range of the generated growth curves. All values were normalized to the growth control (only medium, no hop extract). **MIC_50_** (minimum concentration of a substance that inhibits the growth rate by 50% of the bacteria tested) values were calculated by non-linear regression using GraphPad Prism model 'log(inhibitor) vs. response', where the basal response was substracted and the bottom was set to a constant value (0). Means and standard deviations (**SD**s) were calculated from two biological duplicates, each consisting of three technical replicates.

### MTT Assay for *in vitro* cytotoxic activity

The aqueous hop extracts were investigated for their cytotoxic effect on the chicken cell line UMNSAH/DF-1, using the **MTT** (3-(4,5-dimehtylthiazol-2-yl)-2,5-diphenyltetrazolium bromide) based cell viability assay. Cells were seeded in 96-well microtiter plates (Sarstedt, Nuembrecht, GER) with a cell density of 0.0.05 × 10^6^ cells per mL and 100 µL per well and incubated for 24 h (37°C, 5% CO_2_). Subsequently, the medium was discarded and a dilution series of hop extracts in DMEM was added to the wells (100 µL), ranging from concentrations of 2 mg_BM_ mL^−1^ to 8 µg_BM_ mL^−1^. For vitality control, 20% DMSO (TH Geyer, Renningen, GER) was added, while the growth control received medium only. Treated cells were further incubated for 48 h (37°C, 5% CO_2_). Then, 12.5 µL per well of MTT (Sigma-Aldrich, St. Louis, USA) solution (0.5% (w/v) in phosphate-buffered saline (**PBS**)) was added, followed by another 3 h of incubation (37°C, 5% CO_2_). The plates were centrifuged (300 xg, 5 min), the supernatant was discarded and 20 µL of Igepal (0.4% (v/v) in H_2_O (Sigma-Aldrich, St. Louis, USA) was added. After incubation on a plate shaker for 10 min at 1.000 rpm, 100 µL per well of DMSO was added to dissolve the formazan. The plates were incubated again on the plate shaker for 30 min. Absorbance at 570 nm were measured using a plate reader (PerkinElmer, Waltham, USA). The resulting absorbance was directly proportional to the amount of viable cells. All values were normalized to the growth control. **IC_50_** (concentration of a substance that inhibits the metabolic activity of 50% of the cell line tested) values were calculated analogously to MIC_50_ values. Means and SDs were calculated from two biological duplicates, each consisting of three technical replicates.

### *Ex ovo* shell-less hen’s egg test

To assess biocompatibility, aqueous hop extracts were tested in an *ex ovo* shell-less hen’s egg test on the chick area vasculosa (**HET-CAV**). Fertilized chicken eggs from a local supplier were incubated for 72 h at 37°C and 80% relative humidity. Afterwards, the eggs were cracked into a Petri dish containing Ringer's solution pH 7.0 and 5 g l^−1^ glucose (Carl Roth, Karlsruhe, GER). Only intact eggs in the developmental stage 14 – 17 according to Hamburger and Hamilton (Hamburger and Hamilton, 1992) were selected for the experiments. Samples (10 µL) were pipetted in sterile O-rings made of ethylene propylene diene monomer rubber (**EPDM**) (Kremer GmbH, Wächtersbach, GER) on the chick area vasculosa (**CAV**). A 0.9% NaCl solution (Carl Roth, Karlsruhe, GER) served as negative control, a 1% sodium dodecyl sulfate solution (Carl Roth, Karlsruhe, GER) as positive control, and 2% ethanol as solvent control. The aqueous hop extract from hop pellets were applied in two different concentrations of 10 mg mL^−1^ and 20 mg mL^−1^, while the extract obtained from CO_2_ residue was applied in a concentration of 20 mg mL^−1^. The effects of the extracts on the cardiovascular system, such as bleeding, vascular lysis, blood component aggregation, and cardiac arrest were inspected after 0, 1, 2, 4, 8, and 24 h and images were taken with a Canon EOS 60D camera equipped with an EF-S 18-55 mm lens (both Canon, Tokyo, JPN). The outcome was displayed in a clustergram (Fig. 3), showing the incidence for each effect. Each sample was tested on five eggs in two independent experiments. To ensure validity of the test system, controls were compared to historical laboratory values from previous experiments.

### Microencapsulation of hop extracts into solid lipid microspheres and their characterization

Solid lipid microspheres (**SLM**s) were produced by spray congealing using a custom pilot scale spray congealing apparatus. Three different hop-based SLM formulations were prepared. Each formulation consisted of a matrix material (soy fat, melting point 70°C), 5% (w/w) Nutripur 130 P IP (Cargill, Krefeld, GER) as an emulsifier, 0.5% (w/w) tocopherol (Carl Roth, Karlsruhe, GER), and 1% (w/w) ascorbic acid (Carl Roth, Karlsruhe, GER) serving as antioxidants. To ensure solubility, the ascorbic acid was incorporated as a 20% (w/w) mixture with glycerol (Carl Roth, Karlsruhe, GER). Hop extract (**HE**) of type Herkules, which was gained from industrial-scaled ethanolic extraction (Hopsteiner, Mainburg, GER) was added to formulations to obtain a combined humulone and lupulone content of 1.0% (HE1) or 5.0% (w/w) (HE2 and HE3), respectively. In addition, 1.0% (w/w) XanthoFlav (80% purity, Hopsteiner, Mainburg, GER) was added to HE3 to enrich the xanthohumol concentration. It should be noted that these initial concentrations of bioactive components only reflect the composition of the SLM itself. The concentrations of bioactive compounds in the later-administered feed are much lower, due to the addition of the standard feed.

For production, the matrix material was melted at 80°C. The spray tower was pre-cooled by applying a flow of cool air for 10 min to reach a temperature of at least 15°C. Shortly before microsphere production, the additional components were added to the molten matrix and homogenized using an ULTRA-TURRAX® high-speed stirrer at 10 000 rpm (IKA-Werke, Staufen im Breisgau, GER). The resulting dispersion was maintained at 80°C with a water bath and continuously mixed. The formulation was then pumped using a dosing pump (Drifton A/S, Hvidovre, DNK) into a preheated two-fluid nozzle placed on top of the spray congealing apparatus (DIVA Sprühtechnik GmbH, Hamburg, GER). Solidification of the SLMs was achieved by atomizing the molten formulation into the cooled spray tower using compressed air at 2 bar. An additional counter current airflow of 5 bar was applied at the bottom of the spray tower. The resulting microspheres were separated from the air stream using a cyclone and collected at the end of the process. Particle size distribution was determined by laser diffraction using a Mastersizer 3000 (Malvern Panalytical, Malvern, UK). The particles were dispersed using a dry dispersion unit connected to the measurement chamber.

### Chromatographic analysis of hop extract and SLM composition

The preparation of High Performance Liquid Chromatography (**HPLC**) samples differed for the three types of hop extracts. Ethanolic hop extracts originated from ASE were filtered (0.2 µM, PTFE) and transferred directly to HPLC vials without further preparation. Aqueous hop extracts were 10-fold diluted in mobile phase (see below), incubated for 10 min at 40°C and centrifuged at 10.000 xg for 10 min. Then, 1 mL of supernatant was filtered (0.2 µM, PTFE) and transferred to HPLC vials. Microencapsulated hop extracts (SLMs) were overlaid with methanol in concentrations of 1 mg_capsule_ mL^−1^, 10 mg_capsule_ mL^−1^ and 100 mg_capsule_ mL^−1^ and incubated in an overhead rotator for 1 h at room temperature. After that, 1 mL of filtered (0.2 µM, PTFE) supernatant was transferred to HPLC vials. HPLC measurements were performed according to a previous study (Kober and Castiglione, 2025).

### *In vivo* feeding trials in poultry

The animal model involved 500 *Gallus gallus* (broiler chickens of the COBB 500 lineage). One-day-old chickens were obtained from Avícola Polastri (Itajaí, Brazil). For the experiments, animals were allocated to groups of 20 chickens each at one day of age. Two experimental designs were used to study the *in vivo* antimicrobial effects in broiler chickens. In the first experimental design, four groups were established. The control group received only standard feed (Machs Premium do Brazil, BRA), whereas the three treatment groups were supplemented with grounded hop pellet powder of type Hallertauer Magnum (11.8% humulone, 7% lupulone and 0.5% xanthohumol) (Erzinger et al., 2021), at concentrations of 7%, 10%, or 15% (w/w) in the feed. Supplementation was administered for 45 days from the first day of life.

The second experimental design used the same methodology, the control group was fed only standard feed (Machs Premium do Brazil, BRA); the other groups were supplemented with 6 g per kilogram of feed for 45 days, using the respective SLM batches HE1, HE2 and HE3; one additional group was supplemented with 150 g per kilogram grounded CO_2_ extraction residue powder. The experiment was conducted in the rural area of the municipality of Massaranduba (Brazil), at the poultry facilities located at Estrada Geral Primeiro Braço do Norte, 19130. For sample collection, Cary-Blair Medium Swabs were used, a sterile kit for collecting and transporting clinical samples, especially feces, and preserving pathogens such as *Salmonella, Shigella*, and *Campylobacter* until they reach the laboratory. Samples were collected directly from the chicken cloacas after 45 days of the experiment.

### Microbiome analysis in poultry by MALDI-TOF MS

To determine microbiota composition by Matrix-Assisted Laser Desorption/Ionization Time-of-Flight Mass Spectrometry (**MALDI-TOF MS**), individual colonies were selected from each plate at each dilution step. Two types of culture media were used. MacConkey culture medium is a selective and differential medium for the isolation of Gram-negative bacteria, such as enterobacteria, and for distinguishing between those that ferment lactose and those that do not. It inhibits the growth of Gram-positive bacteria using bile salts and crystal violet. It differentiates Gram-negative bacteria by lactose fermentation, which changes the pH indicator (neutral red) from pink (fermenters) to colorless (non-fermenters). SS Culture Medium (Salmonella-Shigella Agar) is a selective and differential medium, fundamental in microbiology for isolating and identifying bacteria of the genera *Salmonella* and *Shigella* from clinical and food samples. It inhibits Gram-positive bacteria and differentiates microorganisms by lactose fermentation and hydrogen sulfide (H₂S) production. It contains bile salts, brilliant green, and lactose, forming dark colonies (if H₂S is produced, like *Salmonella*) or colorless or pink colonies (if lactose is fermented, like *E. coli*). After 24 hours of incubation, individual colonies were selected for identification. Mass spectra were generated by a Microflex LT MALDI-TOF mass spectrometer (Bruker Daltonics, Bremen, GER), equipped with a nitrogen laser (λ = 337 nm) operating in linear positive ion detection mode, under Biotyper 2.0 automation control (Bruker Daltonics, Bremen, GER).

For the determination of bacterial growth, the semi-quantitative method described by Serena et al. (Serena et al., 2021) was used, which describes the following parameters: no (1), occasional (2), light (3), moderate (4), and heavy (5) growth. For statistical analyses, the software SigmaPlot v. 14.5 (Grafiti LLC, USA) was used. Statistical analysis was performed using one-way ANOVA followed by Tukey's post hoc test. Differences were considered statistically significant at *p* < 0.05.

## Results

### Composition of ethanolic and aqueous hop extracts

**Fig. 1:**
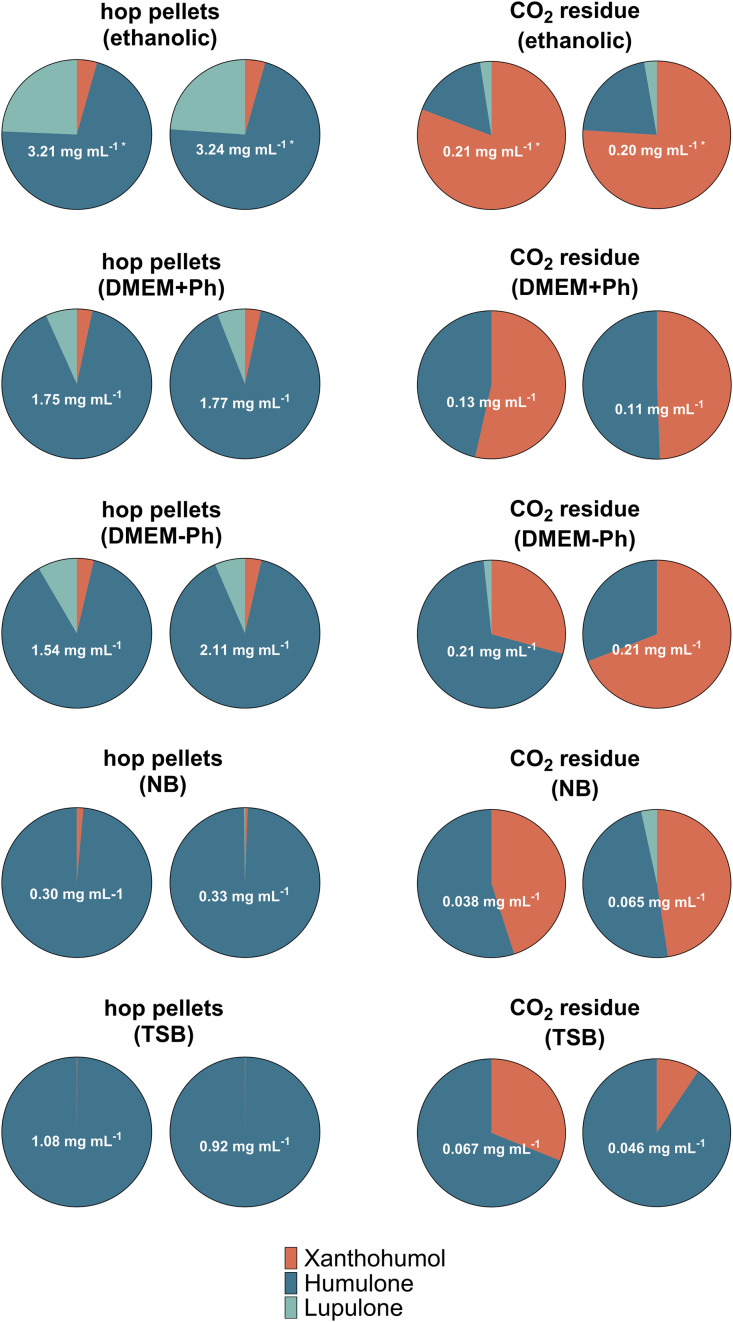
Composition of the ethanolic and aqueous hop extracts with regard to the three main hop isolates xanthohumol, humulone, and lupulone, as well as their combined total concentration. For the ethanolic extracts (*), this refers to a volume of 30 mL immediately after accelerated solvent extraction. In contrast, the aqueous extracts were adjusted to 20 mg mL^−1^ in terms of dried biomass, with the volume used amounting to 13 mL (hop pellets) and 7 mL (CO_2_ residue), respectively. Aqueous extracts were resolubilized in Dulbecco’s Modified Eagle Medium (DMEM) with and without Phenolred (Ph), nutrient broth (NB) and tryptic soy broth (TSB). All extracts occur as biological duplicates.

In order to better assess the results obtained later in *in vitro* tests, the composition of the various extract types must be examined in more detail. The distinct compositions are displayed in Fig. 1. First and foremost, the different raw materials used, namely hop pellets and CO_2_ residue, must be considered. Hop pellets, such as those used in brewing beer, reflect the ingredients of the hop cone itself (Hopsteiner, 2023). Unsurprisingly, the extracts made out of hop pellets mainly contain humulones, but also lupulones and a comparatively low proportion of xanthohumol. During extraction of those hop pellets with supercritical CO₂, humulones and lupulones are primarily extracted, which is why the residual material mainly contains of xanthohumol, but also traces of the other substance classes (Fischer et al., 2025; Rutnik et al., 2021). The extracts obtained in our study reflect this as well. In addition, this also explains the different total concentrations of the combined hop ingredients, as the concentration in the extract of hop pellets is approximately 16 times higher than the one originating from CO_2_ residue.

Furthermore, the compositions differ on the one hand due to the use of organic or aqueous solvents, and on the other hand due to the individual aqueous solvents themselves. Due to the hydrophobic properties of hop compounds, their solubility in aqueous media is generally known to be lower than in ethanolic ones, and even within different aqueous media themselves, the compounds are soluble to varying degrees. Since the total concentration of hop isolates in aqueous CO_2_ residue extracts is comparatively low, it is more difficult to reproduce the results, which leads to slight differences in the composition that are important to consider for subsequent *in vitro* tests.

### Antibacterial and cytotoxic activity of aqueous hop extracts *in vitro*

**Fig. 2:**
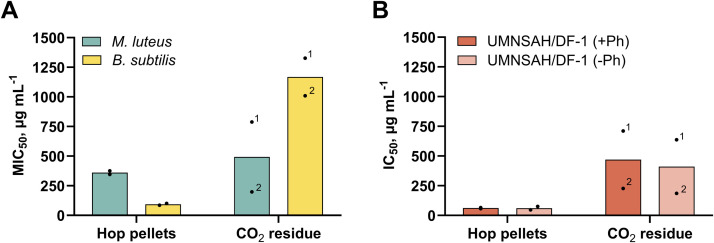
Antibacterial (A) and cytotoxic (B) activity of aqueous hop extracts *in vitro* against bacterial strains Micrococcus luteus and Bacillus subtilis and chicken embryo cell line UMNSAH/DF-1. Mean MIC_50_ and IC_50_ values were displayed as biological duplicates. Extracts obtained out of CO_2_ residue are marked to identify first (1) and second (2) replicate for better classification of trends.

The antibacterial effect of both aqueous hop extracts, made out of hop pellets or CO_2_ residue, was demonstrated in an *in vitro* broth dilution assay (Fig. 2A), which showed that both types of extract were antibacterial effective against the strains *M. luteus* and *B. subtilis*. However, to better classify the different MIC_50_ values gained, the composition of the extracts (Table 1, Fig. 1) must also be considered. Resolubilization of hop pellet extract in TSB resulted in approximately three times the amount of hop compounds being present in NB, may explaining a stronger antibacterial effect on *B. subtilis*. Conversely, the composition and concentration of the extract made from CO₂ residue were relatively similar in both types of medium, yet this extract was approximately twice as effective against *M. luteus* as against *B. subtilis*. The differences in CO₂ extract replication pointed out before are reflected here by higher deviations, but the same trends were observed in both experiments (1 and 2).

On the other hand, the cytotoxic effects of aqueous hop extracts must also be considered (Fig. 2B). The extracts were resolubilized in two different modifications of DMEM. The medium typically recommended and used for the cell line has phenol red added as a pH indicator. However, this red dye could lead to visualization problems in the subsequent *ex ovo* test, as it could interfere with the red colour of the blood vessels. Even though the composition (Fig. 1) had not differed deeply for DMEM with (+Ph) and without (-Ph) phenol red, the cytotoxic effects of both extracts were tested *in vitro*. Firstly, no differences in cytotoxic effects could be detected for the two media types used, which is demonstrated by similar IC_50_ values (Fig. 2B). However, as previously described for antibacterial effects as well, the extracts from hop pellets showed stronger effects, which were represented by lower IC_50_ values than the extracts made from CO_2_ residues. Solvent controls (2% (v/v) EtOH) were included in both assays, which showed neither antibacterial nor cytotoxic effects.

### Biocompatibility of aqueous hop extracts in *ex ovo* hen’s egg tests

Two aqueous hop extracts were assessed in a hen’s egg test on the chick area vasculosa. Pre-incubated fertilized eggs were transferred into petri dishes before the extracts were locally applied on the CAV in a ring system to avoid uncontrolled spreading. Effects like bleeding, vascular lysis, blood component aggregation, and cardiac arrest were inspected for up to 24 h, and the percentage of affected eggs was calculated.

**Fig. 3:**
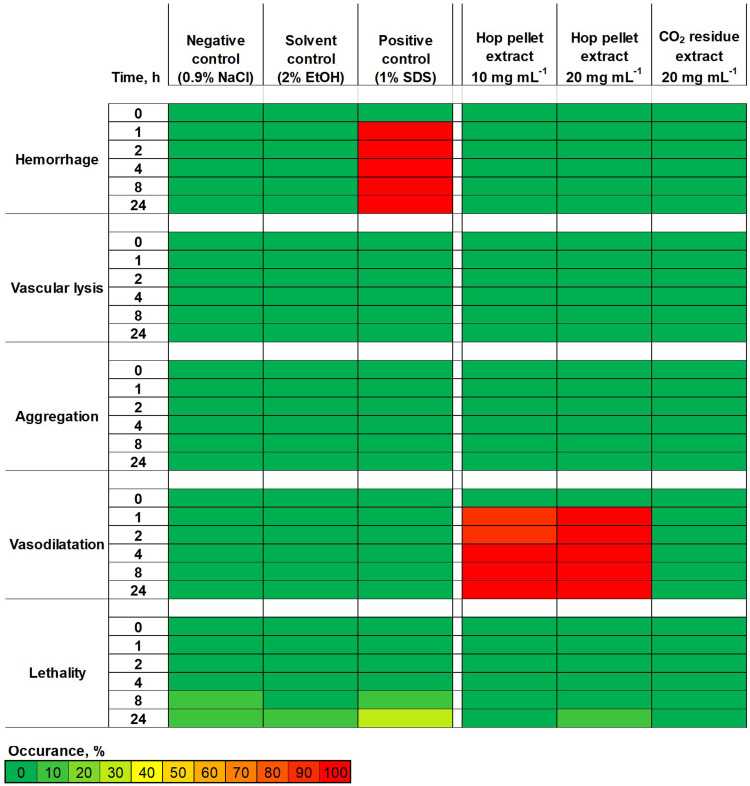
Clustergram showing the time-dependent changes (columns) of the locally applied controls and hop extracts over 24 h in the shell-less hens egg test. The mean percentage of observed effects correlates with the color of the squares, from 0% in green to 100% in red. Each sample was tested in two independent experiments with a minimum total number of 10 eggs. (For interpretation of the references to color in this figure legend, the reader is referred to the web version of this article.).

**Fig. 4:**
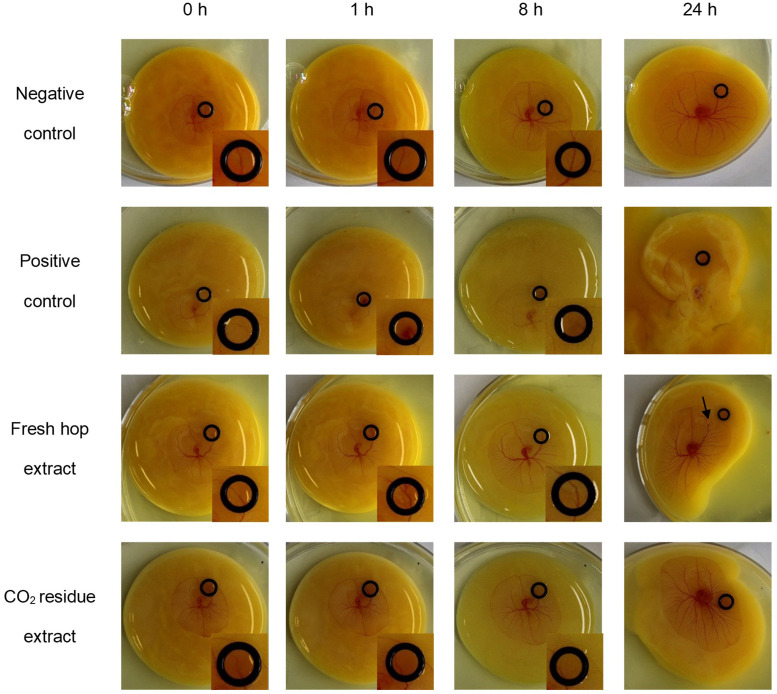
Representative images of observed hen’s eggs with samples of hop pellet extract and CO_2_ residue extract in comparison to the negative and positive control at different time points.

In the negative and solvent control group no effects could be seen, except for one cardiac arrest in each group, which is in agreement with the historical laboratory values. The positive control caused bleeding in all eggs and resulted in three cardiac arrests, which is also consistent with our historical laboratory data and confirmed the validity of the tests. Independent of the tested concentration, the hop pellet extract demonstrated a high biocompatibility without any of these changes, therefore being comparable to the negative control. However, almost all eggs (10/10 for the concentration of 20 mg mL^−1^ and 9/10 for the concentration of 10 mg mL^−1^) revealed an additional effect, showing an enlargement of the blood vessels at the site of local application within the first few hours with a recovery to normal size after 4 to 8 hours. In contrast, the CO_2_ extract showed no effect resembling the negative control (Figs. 3, and 4).

### Characterisation of encapsulated hop extracts (SLMs)

Since three different batches were fed to the chicken, particle size was also measured to rule out the possibility that differences in particle size might lead to variations in uptake by the chicken and thus distort the results regarding efficacy. The volume based median particle diameters (x_50,3_) for HE1, HE2 and HE3 were 158.39 ± 13.62 µm, 110.09 ± 3.58 µm and 75.05 ± 2.17 µm respectively. Poultry feeding behaviour was unaffected by the differences in the size of the particles. All batches of SLMs were completely consumed along with the basal diet.

HPLC measurements were performed for HE3 in order to compare the intended composition with the actual substance concentration after spray congealing of SLMs. Due to the addition of xanthohumol in its pure form, HE3 has the most complex composition and highest concentration of active ingredients, which is why the results obtained here can be transferred to the other two batches, HE1 and HE2. As can be seen in Table 2, the target concentrations of the active ingredients humulone, lupulone, and xanthohumol were achieved. Accordingly, the total concentration of 5% combined humulone and lupulone could also be obtained.

**Table 2:** Targeted and actual compound composition in SLMs (solid lipid microspheres) batch HE3.

Concentration, %	Humulone	Lupulone	Xanthohumol
Targeted	3.71	1.29	1
Actual	3.65 ± 0.18	1.45 ± 0.21	1.01 ± 0.093

### *In vivo* studies of encapsulated hop extracts (SLMs) in poultry

The gastrointestinal tract of birds represents a complex ecosystem, densely colonized by microorganisms that interact dynamically with the host and dietary nutrients. The composition of the intestinal microbiota can vary significantly depending on the rearing system used (Mancabelli et al., 2016). Comparative studies between broiler chickens raised in intensive systems and free-range chickens kept in semi-wild conditions have demonstrated significant differences in microbial community structure. Analyzing the two experimental designs used, it can be observed that the feeding of pure hop pellets - despite having a high concentration of humulone and lupulone (11.8% and 7%) - did not show any significant results between groups, neither in microorganism control nor in terms of mass gain.

**Fig. 5:**
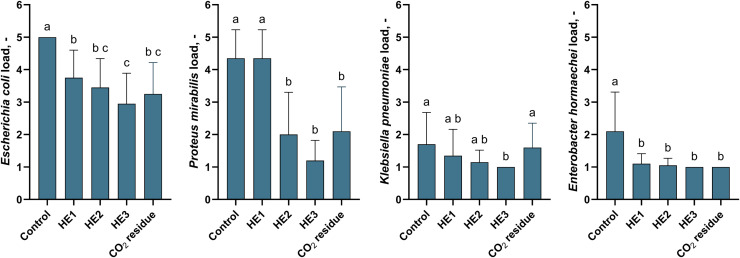
Enhanced antimicrobial effect of SLM batches HE1, HE2, HE3 and CO_2_ residue against *Escherichia coli, Proteus mirabilis, Klebsiella pneumoniae* and *Enterobacter hormaechei* present in the intestinal microbiota of broiler chickens compared to control group without further supplementation. Data are expressed as mean ± standard deviation (SD) (*n* = 20). Different superscript letters indicate significant differences according to Tukey’s HSD test (*p* < 0.05).

The second experimental study, which utilized SLMs showed significant changes compared to the control group, specifically a significant reduction in Gram-negative bacteria, as shown in Fig. 5. These statistical differences are directly related to the concentrations of humulone and lupulone, and are potentiated by the addition of xanthohumol. In terms of mass gain, the results were similar, with a slight mass gain for HE3, but without statistical significance. The control group reached a final weight of 2916 ± 240 g, HE1 reached 2932 ± 313 g, HE2 reached 2952 ± 323 g, HE3 reached 3122 ± 372 g, and the group supplemented with CO₂ residue reached 2925 ± 307 g.

## Discussion

### Selective effects of aqueous hop extracts *in vitro*

In this context, the term ‘selective effect’ refers to the relation between cytotoxic and antibacterial activity. In an ideal scenario, the antibacterial activity would exceed the cytotoxic effect several fold. This can be represented by the ratio of IC_50_ to MIC_50_, where a higher value indicates greater selectivity. However, merely dividing these two values is not a viable approach in the present study. In contrast to studies on isolated compounds (Christensen et al., 2021; Kober and Castiglione, 2025), several factors must be considered when evaluating the effects of whole hop extracts. Firstly, it is important to note that the fundamental composition and total quantity of substances present in the raw material differ substantially. Consequently, the resulting ethanolic and aqueous hop extracts also differ considerably in their compositions. Furthermore, the solubility of the compounds in the respective bacterial and cell culture media can influence the observed activities. Moreover, it is essential to note that, in addition to the hop isolates investigated, other hop compounds are also likely present in the extracts. Although the investigated components are considered the primary contributors to antibacterial efficacy (Kiofentzoglou et al., 2025), there is a lack of research regarding their cytotoxic effects. Finally, it can be said that all substances may also exhibit interaction effects (Kober et al., 2026) within whole hop products, which can be classified as either synergistic or antagonistic (Heinrich et al., 2022). Nevertheless, it is possible to draw two conclusions from the *in vitro* experiments with aqueous extracts, if all the relevant factors are taken into consideration. The analysis indicates that both variants of hop extracts, whether from hop pellets or CO_2_ residue, demonstrate promising properties regarding their utilization as an alternative to AGPs. However, it should be noted that the lower concentration of hop isolates in the residue could potentially necessitate higher application amounts in the future. The differing degrees of effectiveness observed on the two bacterial strains can be attributed to their divergent reactions to the individual hop isolates. Earlier studies demonstrated that xanthohumol was predominantly responsible for the antibacterial effect on *M. luteus*, while the growth rate of *B. subtilis* was also inhibited by humulones and lupulones (Kober and Castiglione, 2025).

### *In vitro* vs. *ex ovo*: cytotoxic activity and biocompatibility of aqueous hop extracts

For an initial assessment, the evaluation of the activity with regard to *in vitro* cytotoxicity is acceptable and provides an important foundation on which further tests can and should be based. Although correlation data exists between *in vitro* and *in vivo* activities for some test systems, this cannot be applied universally. Clearly, the strength of the correlation depends heavily on the organism being considered (Garle et al., 1994). To bridge the gap between *in vitro* and *in vivo* data, we examined an *ex ovo* model (Tahara and Obara, 2021; Warncke et al., 2020), which will enable a three-dimensional cross-model study (*in vitro, ex ovo*, and *in vivo*) for the hop-based feed additives tested within our study. The two aqueous hop extracts tested showed comparatively low IC_50_ values of 75 µg mL^−1^ (fresh hop extract) and 400 µg mL^−1^ (CO_2_ residue extract) *in vitro*, while the *ex ovo* test showed only signs of biocompatible behaviour up to concentrations of 20 mg mL^−1^. Accordingly, the extracts were less harmful in *ex ovo* testing, respectively, suggesting that such effects *in vivo* should also be significantly lower than *in vitro* and that hop based feed additives should be biocompatible with the chicken organism. Furthermore, eggs with hop pellet extract applied showed enlarged vessels within the first few hours of testing. This enlargement could be due to vasodilatative effects, which are described in literature for hop extract components such as humulones (Di Pietro et al., 2024; Figard et al., 2008). This effect didn’t occur for eggs tested with CO_2_ residue extract, which can be forwarded to the lower concentrations of present bioactive hop components. Improved vasodilation in chickens may have positive effects, such as supporting thermoregulation through changes in blood flow (Nolan et al., 1978).

### *In vivo* studies of encapsulated hop extracts in poultry

Hop-derived bitter acids exhibit well-documented antimicrobial activity, particularly against Gram-positive bacteria (Bocquet et al., 2019). Additionally, the negative outcome of the first study with grounded hop pellets may be justified by the likely inability of the broiler chickens' digestive systems to extract hop resins efficiently. The results of the second study are therefore even more encouraging, since in this study, using whole hop extracts in microencapsulated form as SLMs, the results on Gram-negative bacteria differ from those previously reported in the literature, where these hop resins show lower efficiency against Gram-negative bacteria than against Gram-positive bacteria. This fact may be justified by the microencapsulation, which used saturated fats as a vehicle, and these may have facilitated their entry into the lipid membrane of Gram-negative bacteria, given the significant reduction in the microbial load observed for the four bacteria identified in the biota of broiler chickens. Notably, the SLM formulation HE3 enriched with xanthohumol exhibited the most pronounced antimicrobial effects, suggesting that prenylated flavonoids make a particularly strong contribution to the observed activity. These findings are consistent with recent studies reporting the beneficial effects of plant-derived polyphenols on the gut health of broiler chickens through dietary tannins (Liu et al., 2023; Sun et al., 2026). This supports the functional relevance of polyphenolic compounds in poultry nutrition. In addition, *in vitro* studies on plant-derived polysaccharides, which may be partially extracted due to hydroethanolic mixtures, have demonstrated that these compounds can modulate gut microbial fermentation activity, thereby further supporting the general role of plant bioactives in shaping intestinal microbiota (Chen et al., 2025; Li et al., 2022).

In the small intestines of poultry of zootechnical interest, microorganisms such as *Lactobacillus* spp., *Enterococcus*, and members of the *Clostridiaceae* family predominate. *Lactobacillus* spp. play an essential role in maintaining intestinal homeostasis, stimulating the production of immunoglobulins and the synthesis of metabolites such as lactate and acetate. These compounds favour the proliferation of beneficial bacteria, such as *Bacillus* spp., *Bacteroides* spp., and *Bifidobacterium* spp., which produce volatile fatty acids. The production of these acids helps reduce intestinal pH, creating an environment unfavourable to colonization by pathogenic microorganisms. Additionally, the beneficial microbiota competes for adhesion sites on the intestinal mucosa, hindering the establishment of agents such as *E. coli, Campylobacter* spp., and *Salmonella* spp., all members of the Enterobacteriaceae family. *E. coli* is a Gram-negative bacterium that can act as an intestinal commensal; however, certain strains exhibit opportunistic behaviour and pathogenic potential (Cruz et al., 2022). The high adaptability of *E. coli* enables its colonization and persistence in the intestinal tract and its ability to evade the host's immune defences. However, its excessive multiplication poses a health risk to both poultry and public health, potentially leading to meat contamination and foodborne illnesses in humans. Therefore, reducing *E. coli* bacterial load, especially in the cloacal region, is a relevant measure to minimize transmission risks and enhance the safety of poultry products (Christensen et al., 2021).

The detection of Gram-negative *Proteus mirabilis, Klebsiella pneumoniae*, and *Enterobacter hormaechei* in the intestinal flora of broiler chickens is consistent with previous findings indicating that members of Enterobacteriaceae are common constituents of the avian gut microbiota (Pace et al., 2025), albeit typically at low relative abundances. In a balanced intestinal ecosystem, the microbiota is dominated by Bacillota, particularly lactic acid–producing bacteria, which contribute to gut health, nutrient absorption, and competitive exclusion of pathogens (Sun et al., 2022). In contrast, an increased representation of opportunistic Gram-negative bacteria, such as *P. mirabilis, K. pneumoniae*, and *E. hormaechei*, is often interpreted as a shift toward dysbiosis. Several factors may contribute to this microbial imbalance, including dietary composition, environmental contamination, stocking density, physiological stress, and antimicrobial use. Under suboptimal conditions, these genera may proliferate and outcompete beneficial microbiota, potentially leading to impaired intestinal barrier function, increased inflammation, and reduced growth performance. *E. coli* is a bacterium that can act as an intestinal commensal; however, certain strains exhibit opportunistic behaviour and pathogenic potential (Cruz et al., 2022).

## Conclusion

Avian pathogenic infections are associated with significant economic losses and reduced animal welfare. In broiler production, prophylactic use of antibiotics as AGPs led ultimately to a higher prevalence of antibiotic-resistant *E. coli, P. mirabilis, E. hormaechei*, and *K. pneumoniae*. There are concerns that the transfer of these antibiotic-resistant microorganisms through the food chain may pose risks of extraintestinal infection in humans, through zoonotic transmission, and greater difficulties in treating human infections caused by these bacteria. This study highlights the potential of hop extracts as phytogenic feed additives for reducing antimicrobial resistance in poultry production, when used as PFA. By combining *in vitro, ex ovo* and *in vivo* analyses, the bioactive efficacy, biocompatibility, and safety of hop-derived extracts were comprehensively assessed. Two hop-based product formats, namely microencapsulated hop pellet extracts as solid lipid microspheres (SLMs) and direct use of CO₂ residue, proved particularly effective, showing antimicrobial effects with low cytotoxicity. In animal models, they led to a significant reduction in Gram-negative intestinal bacteria without negatively affecting performance. Importantly, while previous studies have largely focused on the effect of hop derivatives on Gram-positive bacteria, the present findings demonstrate effects on Gram-negative intestinal microbiota in broilers. Using bioactive hop components via microencapsulation is thus a practical and highly relevant alternative for health protection in broiler production. The direct use of CO₂ residues, which are less susceptible to oxidation than whole hop sources, also proved promising, although at a presumably higher application amount. Future studies should investigate the long-term effects on the gut microbiome and possible immunomodulation, as well as optimising microencapsulation to fine-tune release and absorption characteristics.

## Declarations

### Ethics approval and consent to participate

This study was approved by the Research Ethics Committee of the University of the Joinville Region (UNIVILLE; protocol number 006/2023). All experimental procedures involving animals were conducted in accordance with institutional and national guidelines for the care and use of animals in research, and all efforts were made to ensure animal welfare throughout the study. The study involved standard feeding practices and did not include invasive procedures.

### Consent for publication

Not applicable.

### Availability of data and materials

All data generated and analysed during this study are included in this published article. Further data is available from the corresponding author on reasonable request.

### Funding

This study was funded by the German Bundesministerium für Forschung, Technologie und Raumfahrt (BMFTR, Grant-No. 031B1253) and the Financiadora de Estudos e Projetos (FINEP) on behalf of the Ministério da Ciência, Tecnologia e Inovações, Brazil.

## CRediT authorship contribution statement

**Luisa Kober:** Writing – review & editing, Writing – original draft, Visualization, Validation, Project administration, Methodology, Formal analysis, Data curation, Conceptualization. **Sebastian M. Strauch:** Writing – review & editing, Writing – original draft, Data curation, Conceptualization. **Veronika Grassl:** Writing – original draft, Visualization, Formal analysis, Data curation. **Monique Fröhlich Tonet:** Writing – original draft, Formal analysis, Data curation. **Nora Hirschmann:** Writing – original draft, Formal analysis, Data curation. **Dagmar Fischer:** Writing – review & editing, Writing – original draft, Supervision. **Gilmar S. Erzinger:** Writing – review & editing, Writing – original draft, Supervision, Funding acquisition. **Kathrin Castiglione:** Writing – review & editing, Writing – original draft, Supervision, Project administration, Funding acquisition, Conceptualization.

## Disclosures

The authors declare no competing interests.
